# Behavioral activation for depression in patients with advanced cancer: study protocol for a multicenter randomized controlled trial

**DOI:** 10.1186/s12885-023-10926-y

**Published:** 2023-05-11

**Authors:** Takatoshi Hirayama, Yuko Ogawa, Asao Ogawa, Emi Igarashi, Saaya Soejima, Kotone Hata, Yusuke Utsumi, Yuki Mashiko, Kyoka Ogata, Ayako Kayano, Yuko Yanai, Shin-ichi Suzuki

**Affiliations:** 1 Department of Psycho-Oncology, National Cancer Center Hospital, 5-1-1 Tsukiji, Chuo-ku, Tokyo, Japan; 2 Department of Psycho-Oncology, National Cancer Center Hospital East, 6-5-1 Kashiwanoha, Kashiwa-shi, Chiba Japan; 3 Department of Psychiatry, Graduate School of Medicine, Tohoku University, 2-1 Seiryo-machi, Aoba-ku, Sendai, Miyagi Japan; 4 Faculty of Human Sciences, Waseda University, 2-579-15 Mikajima, Tokorozawa-shi, Saitama Japan; 5 Department of Psychiatry, Tohoku University Hospital, 1-1 Seiryo-machi, Aoba-ku, Sendai, Miyagi Japan

**Keywords:** Behavioral activation, Depression, Advanced cancer, Psychotherapy, Psycho-oncology, Randomized controlled trial, Study protocol

## Abstract

**Background:**

Though behavioral activation (BA) has been shown to be effective for depression, evidence in patients with advanced cancer has not been established. This study aimed to examine the effectiveness of a BA program on depression in this population.

**Methods:**

A randomized controlled trial with a wait-list control group (waiting group) of 38 patients with advanced cancer and depression will be conducted at three sites in Japan. The BA program consists of seven sessions. Outcome measures will be evaluated at three times in the intervention group; at the entry, at the end of the intervention and 4 months after the end of the intervention and four times in the waiting group: at the entry, before the intervention, at the end of the intervention, and 4 months after the end of the intervention. Primary outcome is Beck Depression Inventory-II (BDI-II) score. To examine the main effect of the intervention, two-way repeated measures analysis of variance (ANOVA) will be conducted, with timing and intervention status as the independent variables and BDI-II score as the dependent variable. One-way repeated measures ANOVA will be conducted to combine data from the intervention and control groups and examine changes in BDI-II scores by timing in both groups. Secondary endpoints (anxiety, quality of life, spirituality, degree of behavioral activation, value, and pain) will be evaluated with rating scales. Two-way repeated measures ANOVA will be conducted to examine whether there are differences between the groups before and after the intervention, with timing and intervention status as the independent variables and scores on each rating scale as the dependent variables.

**Discussion:**

This multicenter randomized controlled trial is the first study to assess the effectiveness of BA on depression in patients with advanced cancer. Our findings will provide evidence about the effectiveness of BA on depression and provide an intervention option that is acceptable and feasible for the treatment of depression in this population. The results of this study will lead to improved mood and rebuilding to regain life purpose and value in this vulnerable population.

**Trial Registration Number:**

jRCT, jRCT1030210687, Registered 22 March 2022, https://jrct.niph.go.jp/en-latest-detail/jRCT1030210687.

**Supplementary Information:**

The online version contains supplementary material available at 10.1186/s12885-023-10926-y.

## Background

Patients with advanced cancer have unmet needs in the financial, health system, health information, psychological, physical, and daily living domains. These unmet needs are associated with distress, depression, and anxiety in patients with advanced cancer [1]. They also experience existential distress as a result of a shortened lifespan, prognostic uncertainty, altered interpersonal relationships, and impaired physical functioning, sense of autonomy, and personal control [2]. Previous studies have reported that psychological distress, particularly depression, is frequently observed in patients with cancer, especially advanced cancer [3–6].

Depression in patients with advanced cancer leads to adverse effects such as impaired quality of life (QOL) [7], risk of suicide [8], and psychological burden on their family [9, 10]. Therefore, an important challenge in clinical oncology is determining which interventions are most acceptable and feasible to treat depression in patients with advanced cancer [11].

There is a general preference for psychotherapy in the most common psychiatric disorders, but also specifically for patients with cancer [12, 13]. Meaning-based individual psychoeducational interventions [14] and legacy-based interventions have been found to be beneficial for individuals near the end of life [15]. However, there are few brief evidence-based psychotherapies that are simple to deliver and integrated with cancer care from the time of diagnosis to advanced disease [16, 17]. Previous studies have not shown consistent results regarding effectiveness; many methodological problems have been pointed out [11].

Patients living with advanced cancer experience various types of psychological and social distress such as anxiety about worsening disease, fear of death, and hopelessness about life, which often lead to mental health problems such as depression and high risk of suicide [18]. Quite a few patients with cancer are reluctant to directly face their concerns regarding death, symptom burden, loss of control, and other factors because their experiences are psychologically traumatic [19]. Several studies have shown the effectiveness of cognitive restructuring-based cognitive behavioral therapy (CBT) for depression or anxiety in patients with advanced cancer [20, 21], while other studies have reported no effectiveness [22]. Evidence about the effectiveness of CBT in patients with advanced cancer is inconsistent.

Behavioral activation (BA), developed as a psychotherapy for depression, aims to improve mood and rebuild life to regain purpose and value through the reuse of the patient’s inherently healthy aspects as well as human and social resources, rather than addressing the pathology of depression and negative thoughts and feelings, which had been the focus of previous psychotherapies. Many studies have demonstrated the effectiveness of BA in depression with a high level of evidence [23]. Some studies have evaluated the use of BA in patients with breast cancer or cancer-free survivors [24, 25]. However, no studies have explored the effectiveness of BA in patients with advanced cancer.

To explore the applicability of BA to patients with cancer, we have developed a BA program for patients with cancer and tested the preliminary effectiveness in a pre-post study without a control group [26]. This program was designed to (1) understand confining life patterns through activity records, (2) discuss specific activity plans and devise ways to implement them, (3) focus on positive aspects such as the joy and sense of accomplishment associated with activities, (4) clarify what is important to the patient as well as the joy and value of life, (5) break free from pathological anxiety and worry, and (6) create a life plan for the future. The program consists of seven sessions. The preliminary study showed high rates of treatment completion and remission of depressive symptoms, regardless of the stage of cancer, suggesting that BA could be a potentially effective psychological intervention for patients with advanced cancer [26].

This multicenter randomized controlled trial aims to examine the effectiveness of a BA program on depression in patients with advanced cancer compared to a wait-list control group (waiting group).

## Methods

### Study setting

Participants will be recruited from May 2022 to March 2024 by the Department of Psycho-Oncology at the National Cancer Center Hospital, the Department of Psycho-Oncology at the National Cancer Center Hospital East, and the Department of Psychiatry at Tohoku University Hospital, Japan.

### Patient and public involvement

Based on feedback from patients who participated in the pilot study that the program contents were very useful but that shorter sessions would be more accessible [19], this BA program [26] was modified to include seven instead of eight sessions. Patients or the public will not be involved in the design, reporting, or dissemination plans of this study.

### Eligibility

#### Inclusion criteria

All study participants will meet the following criteria: (1) diagnosis of cancer or recurrence for more than 1 month with clinical stage III or IV disease for which the attending physician has determined that radical cure is not a goal, based on confirmation from the medical record or the attending physician directly; (2) expected prognosis of approximately 1 year or more according to the attending physician; (3) ability to participate in all sessions; (4) depression, defined as 16 ≥ points on the Beck Depression Inventory-II (BDI-II) (regardless of whether or not they meet the diagnostic criteria for depression); (5) age of 20–64 years, or age of ≥ 65 years with ≥ 24 points on the Mini Mental State Examination-Japanese (MMSE-J) ; (6) Eastern Cooperative Oncology Group (ECOG) performance status (PS) of 0 or 1 according to the attending physician; (7) ability to speak Japanese; and (8) written informed consent to participate in this study and wish to continue with the intervention.

#### Exclusion criteria

The exclusion criteria will be as follows: (1) severe physical or psychological symptoms (cognitive dysfunction [defined as a score of ≤ 23 on the MMSE-J performed for all prospective participants aged ≥ 65 years or those aged 20–64 years who did not seem to understand routine instructions during the preliminary interview], impaired consciousness, severe depression with psychotic symptoms, imminent suicidal ideation, and history of suicide attempt); (2) receipt of psychological intervention, such as BA, by mental health professionals before enrollment; (3) use or planned use of any psychotropic drug during the program, except for temporary emergency use after enrollment based on the discretion of the attending physician; (4) substance dependence based on the diagnostic criteria in the Diagnostic and Statistical Manual of Mental Disorders, 5th edition; and (5) difficulty in participating in this program due to other reasons, according to the attending physician or researchers.

The waiting group will be informed that if the need for medication arises during the waiting period, they will be excluded from the study but can be offered another program equivalent to the BA program if they wish.

#### Discontinuation criteria

The discontinuation criteria will be as follows: (1) serious adverse events or adverse reactions that make it difficult to continue the intervention; (2) worsening of physical or psychiatric symptoms, or emergence of new symptoms that make it impossible to continue the intervention; (3) patient desire to discontinue the intervention; (4) interval of 1 month or more between sessions attended; (5) regular use of psychotropic drugs at the discretion of the attending physician during the implementation of the program or while waiting; (6) meeting the exclusion criteria while waiting; and (7) any other reason for which the attending physician deems it necessary to discontinue the intervention.

If this study will be discontinued, the date on which the decision for discontinuation was made and the reason for discontinuation will be noted on the case registration form. The data center will be informed of the discontinuation.

If serious adverse events or adverse reactions, such as worsening of psychiatric or physical symptoms, occurs and it is deemed difficult to continue the intervention, the therapist will promptly inform the research director at each research institution and discontinue this study. The research director at each research institution contacted will promptly inform the research representative and data center of the decision for discontinuation.

### Interventions

The program consists of seven 50-minute sessions conducted over 1–2 weeks, with an average of 5–10 minutes of homework per day. The themes and contents of the program are shown in Table 1. Whenever possible, sessions will be held on the same day as examinations or consultations with the attending physician to improve adherence to intervention protocols.

**Table 1:** Themes and contents of the behavioral activation program

Session	Theme	Contents
1	Let’s begin!	• Understand the relationship between emotions and behaviors • Learn tips on how to avoid being preoccupied by cancer in order to regain pleasure and meaning in life
2	Identify the relationship between emotions and behaviors	• Explore the relationship between emotions and daily activities • Identify patterns of anxiety, depression, and calmness
3	Identify activities that make your life pleasurable	• Clarify life values • Identify activities that are achievable in one’s current situation
4	Review the results of activities	• Evaluate the usefulness and difficulty of each activity • Discuss ways to participate in challenging activities
5	Identify difficult situations and understand ways to change one’s feelings	• Identify situations that are likely to lead to anxiety and depression • Identify thoughts that occur in the early stages of anxiety and depression as well as any vicious cycles that might be involved • Identify pros and cons of negative thinking
6	Learn to live a life of value	• Review how the program helped change patterns of daily life • Identify future goals and make plans to achieve them
7	Wrap-up and graduation	• Review what has been learned through the program • Identify future issues and how to address them

This program was verified in a pilot study that explored the applicability of BA to patients with cancer [19]. The pre-post study suggested that the program was feasible and effective for depression in patients with cancer [26].

All therapists will be clinical psychologists or psychiatrists with clinical experience that includes working with patients with cancer and have sufficient experience conducting BA either during the pre-post study [26] or through BA training. A therapist is certified in BA training upon completion of the following three steps: (1) learning about the aims of BA, session structure, and how to proceed by attending an in-person or video training; (2) conducting BA with supervision at least once; and (3) being approved by a supervisor to perform BA.

The individuals responsible for measuring outcomes and assessing patients will differ from those who are responsible for conducting the program. While this program will not impact the patients’ regular treatment in any way, special psychotherapies such as BA (conducted elsewhere) and CBT are prohibited.

### Study procedure

Information about the study will be communicated to each department of each research institution through postings, in-hospital e-mails, websites, and other means. The contact information of the research office will be clearly indicated. Patients who visit each research institution during the study period and wish to participate in the study after recommendation by their attending physician, and those who wish to participate in the study on their own and whose attending physicians determine that their participation is appropriate, will be selected from among those who meet the eligibility criteria and who do not meet the exclusion criteria. For patients who meet the eligibility criteria but not the exclusion criteria, the therapist will provide a detailed explanation during the preliminary interview and obtain their consent to participate in the study. Once consent is obtained, the therapist will confirm the eligibility and exclusion criteria other than the BDI-II score and ask the research assistant to administer questionnaires. The research assistant will ask the participant to fill out the BDI-II questionnaire and transfer the data (entry number, anonymized subject information, and T1 outcome data) to the data center with the entry number written on the case registration form. If the BDI-II score is 16 or higher, the patient will be registered in this study. Once a patient is registered, the registration will not be cancelled (deleted from the database) except in the case of withdrawal of consent, including refusal to use the data for research purposes. In the case of duplicate enrollment, the first enrollment information will be used.

**Fig. 1:**
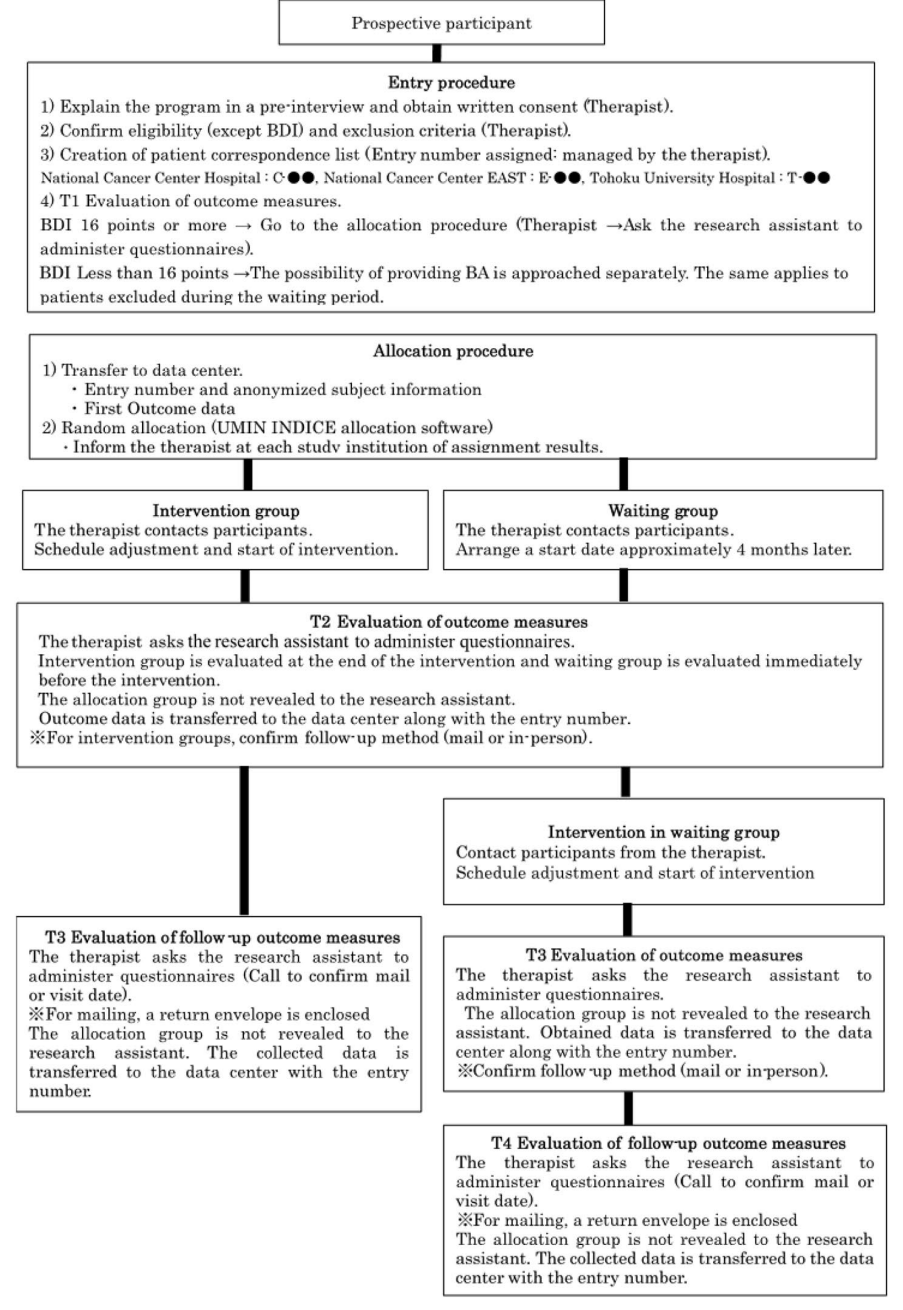
Flow of registration and evaluation for this study. Participants will be randomized 1:1 into the intervention group or the waiting group. Participants will have seven intervention sessions, delivered over 4 months. Outcome measures will be evaluated at three times (T1–T3) in the intervention group and four times (T1–T4) in the waiting group

**Fig. 2:**
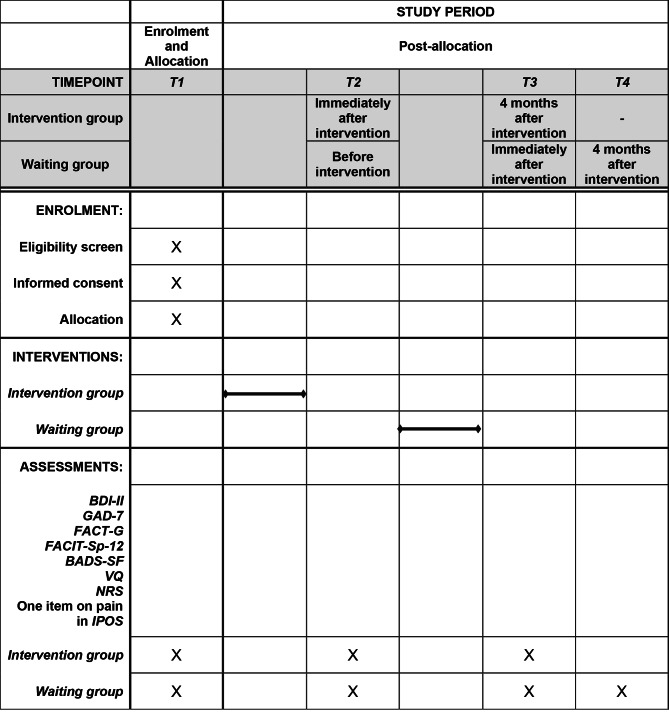
SPIRIT figure: Schedule of enrollment, interventions, and assessments. *BDI-II* Beck Depression Inventory-II, *GAD-7* Generalized Anxiety Disorder-7, *FACT-G* Functional Assessment of Cancer Therapy-General, *FACIT-Sp-12* Functional Assessment of Chronic Illness Therapy - Spiritual Well-Being 12 Item Scale, *BADS-SF* Behavioral Activation for Depression Scale-Short Form, *VQ* Valuing Questionnaire, *NRS* Numerical rating scale, *IPOS* Integrated Palliative care Outcome Scale

The intervention and each assessment scale will be evaluated using the following process (Figs. 1 and 2). The University Hospital Medical Information Internet Data and Information System for Clinical and Epidemiological Research (UMIN INDICE) allocation software program will be used for case enrollment and randomization of intervention groups in this study. Randomization was stratified by study site with the minimization method to balance the gender (male, female) and the age of the participants at study entry (age 20 ≦ to < 40, age < 65, age 65 to < 100). For allocation concealment to research assistants who administer questionnaires, the allocation will be communicated to the person in charge at each site based on the allocation list prepared by the data center. In addition, the interval between T1 and T2, T2 and T3, T3 and T4 will be 4 months respectively, which is the same length of time it would take if the intervention group had implemented the program, so that research assistants will not be able to identify the allocation group based on the timing of evaluations. The allocation schedule will be kept at the data center until the end of the study. A psychiatrist or psychologist from each institution will be in charge of a total of seven intervention sessions. Outcome measures will be evaluated at three times (T1–T3) in the intervention group and four times (T1–T4) in the waiting group by research assistants who are not involved in the intervention program. Outcome measures will include the BDI-II score as the primary outcome measure and Generalized Anxiety Disorder-7 (GAD-7), Functional Assessment of Cancer Therapy-General (FACT-G), Functional Assessment of Chronic Illness Therapy - Spiritual Well-Being 12 Item Scale (FACIT-Sp-12), Behavioral Activation for Depression Scale-Short Form (BADS-SF), the Valuing Questionnaire (VQ), Numerical rating scale (NRS), and the Integrated Palliative care Outcome Scale (IPOS) scores as secondary outcome measures.

Participants in the intervention group will be asked to complete questionnaires at three times (T1–T3). Participants in the waiting group will be asked to complete questionnaires at four times (T1–T4). They will complete questionnaires in the waiting room, at the examination room after the BA session at the facility, or at home. Research assistants will check the responses and ask participants to supply any missing data. The research assistants will collect the completed questionnaires. They will transfer the data from the questionnaires to the data center.

### Outcomes

#### Primary outcomes

The primary outcome is the BDI-II score. The BDI-II is used to measure depressive symptoms. It consists of 21 self-reported items scored on a 4-point scale [27]. The psychometric criteria for the BDI-II are generally considered to be excellent when the instrument is administered to outpatients [27, 28]. Good reliability and validity have been reported for the Japanese version [29]. In order to ensure comparability with previous studies [24, 30, 31], we will use the BDI-II, which is similar to the scales used in previous studies. The BDI-II is widely used worldwide to assess subjective depression. We will compare whether there is a difference in BDI-II scores between the intervention group and the waiting group before and after the intervention.

#### Secondary outcomes

Secondary outcomes consist of the change from the pre-intervention scores to the post-intervention scores on the following psychosocial measures: (1) GAD-7, (2) FACT-G, (3) FACIT-Sp-12, (4) BADS-SF, (5) VQ, (6) NRS, and (7) one item on pain in IPOS.

GAD-7 is a measure used to assess subjective anxiety. GAD-7 is a seven-item questionnaire developed to identify probable cases of GAD and to measure the severity of GAD symptoms [32]. A Japanese version has been validated [33]. The total score for GAD-7 ranges from 0 to 21.

FACT-G is a measure used to assess quality of life [34]. This widely used questionnaire consists of 27 items, with higher scores indicating higher QOL. The questionnaire comprises four domains: physical, social, emotional, and functional well-being.

FACIT-Sp-12 is a measure used to assess QOL that includes spirituality, which is important for patients with advanced cancer in clinical practice. It is a QOL assessment scale consisting of 12 items related to spirituality developed by Cella et al. [35, 36]. The rating scale is on a five-point scale from 0 (not at all applicable) to 4 (very applicable) with higher scores indicating higher QOL. The reliability and validity of the Japanese version have been confirmed [37].

BADS-SF is a measure used to assess the degree of BA and avoidance. BADS-SF was developed to assess changes in behavior resulting from BA [38]. BADS-SF comprises subscales about two traits, activation and avoidance. The Japanese version of this scale consists of eight items, which is one fewer than in the original version. The validity and reliability of the Japanese version have been confirmed [39].

VQ is a measure used to assess whether patients are engaging in value placing behaviors. VQ is a 10-item self-reported questionnaire that measures the degree to which one’s behaviors are consistent with their values [40]. A score of each item in VQ is calculated for each of the two factors, VQ Progress (VQ-P) and VQ Obstruction (VQ-O). VQ-P measures the extent to which individuals are aware of what is personally important to them and their perseverance in achieving whatever that is. VQ-O measures the extent to which living in line with one’s values is disrupted by avoiding experiences that distract from this goal, either due to neglect or a focus on other psychological experiences. The validity and reliability of the Japanese version have been confirmed [41].

NRS is a measure used to assess the degree of pain associated with physical symptoms. NRS asks for a pain score on a scale of 0 to 10, with 0 being no pain at all and 10 being the worst possible pain. It has been used in actual clinical practice and has been validated for both reliability and validity [42, 43].

One item on pain in IPOS assesses the extent to which pain interferes with life. IPOS is a holistic assessment instrument for palliative care that was validated in 2016 by Schildmann et al. The Palliative Care Outcome Scale (POS) [44] was developed in 1999 and its derivatives have been integrated as IPOS. A patient version and a staff version have been developed [45]. The validity and reliability of the Japanese patient version have also been verified [46]. The questionnaire uses a five-point scale from 0 (it did not disturb me at all) to 4 (it was unbearable) for the pain item in the Physical Symptoms section.

### Power and sample calculation

Based on the results of a previous study [47], the minimal clinically important difference of the primary endpoint, BDI-II, is 3.5 points. In our pre-post study of patients with cancer and depression [26], the standard deviation was 7.84 points. Many studies have demonstrated the effectiveness of BA in depression with a high level of evidence [23–25]. We hypothesized an alternative hypothesis that intervention group would have lower mean post-intervention scores on the BDI-II than waiting group. The sample size was calculated using a one-tailed test with α = 0.05, power (1-β) = 0.80, and r = 0.50 for the mean value of the two groups. Since 3 (9.4%) out of 32 participants dropped out of the aforementioned pre-post study [26], we assumed that 10% would drop out in this study as well, defined as patients that did not meet the exclusion criteria and discontinued participation. Thus, the total planned enrollment was set to 38 patients.

### Statistical methods

#### Major statistical analysis

To examine the treatment effect parameters of all randomly assigned subjects in the primary analysis set, we will analyze the primary outcome according to the intention-to-treat principle. The significance level will be 5% and one-sided. The analysis was based on the analysis of a previous randomized, wait-listed, controlled trial of psychotherapy in cancer patients [48]. First, two-way repeated measures analysis of variance (ANOVA) will be conducted to examine the main effect of the intervention based on intervention status, with timing (pre-intervention, post-intervention, and 4 months post-intervention) and intervention status (intervention group and waiting group) as the independent variables and the primary endpoint, BDI-II score, as the dependent variable. Next, one-way repeated measures ANOVA will be conducted to integrate data from the intervention and waiting groups and to examine changes in BDI-II scores by time (pre-intervention, post-intervention, and 4 months after intervention) in both groups. When there is any missing data, we will employ multiple imputation.

#### Secondary statistical analysis

Exploratory secondary statistical analyses will be conducted to provide additional insights to supplement the results of the primary statistical analyses. To examine whether there are differences between groups before and after the intervention for GAD-7, FACT-G, FACIT-Sp-12, BADS-SF, VQ, NRS, and IPOS, there will be two independent variables: timing (pre-intervention, post-intervention, and 4 months post-intervention) and intervention status (intervention group and waiting group). The dependent variable will be the score of each rating scale. Note that for GAD-7, FACIT-Sp-12, NRS, and IPOS, the overall score for each scale will be calculated. For FACT-G, BADS-SF, and VQ, the overall score and subscale scores will be calculated.

#### Interim analyses

We do not plan any interim analyses.

### Ethics and dissemination

#### Research ethics approval and protocol amendments

This study was approved by the Research Ethics Committee of the National Cancer Center Institutional Review Board (approval number, 2021 − 312; protocol version 1.4; January 17, 2022). Modifications to the protocol will be shared promptly at all three participating hospitals. They will be communicated to relevant parties immediately, including study participants, journal editors, and ethics committee members. This study will be conducted in accordance with the Declaration of Helsinki [49] and the Ethical Guidelines for Medical and Health Research Involving Human Subjects [50, 51].

#### Consent or assent

Explanatory documents (See Supplemental materials 1) that describe the study, its objectives, and the potential benefits and risks will be provided to the participants. They will also indicate that withdrawal from the study will not affect their current or future clinical care. However, we will explain to the participants that any results already presented at academic conferences and or in manuscripts before withdrawal of consent will not be retracted. Participants will also be informed that if the study damages their health, they will receive general medical care as compensation. We will recommend that patients inform their family about participation. After eligible patients have signed the consent forms, one copy will be given to the patient and the original will be collected by the therapist.

### Data management and confidentiality

Clinical data (age, gender, education, employment status, marital status, cancer diagnosis, metastasis, cancer stage, history of cancer treatment, current cancer treatment, current medication, psychiatric history, ECOG PS, and questionnaire data) obtained from this study will be managed anonymously by a data manager. All participants will be assigned a unique subject identification number (ID). All data will be coded by ID; all study-related documents will be anonymous. The consent forms containing participants’ signatures and master lists linking participants’ names to ID numbers will be stored in secure servers or in locked office filing cabinets at each research facility. Researchers will analyze the stored data. The principal investigator and researchers who will analyze the data will have access to the final dataset. The results will be submitted for peer-reviewed publication and presentation at local, national, and international scientific meetings and conferences.

Since there are few adverse events expected during this study and it is not an invasive study as defined by the Ethical Guidelines for Medical and Health Research Involving Human Subjects [50, 51], no data monitoring will be conducted in this study.

### Expected advantages and disadvantages

#### Patient benefit

There is a significant association between depression and advanced or metastatic cancer [52]; however, depression tends to be undetected and untreated in this population [53, 54]. There is little evidence that antidepressants are beneficial compared with placebo in patients with advanced cancer [55]. A disadvantage of both antidepressants and anxiolytics in advanced cancer is the substantial side effects, which include seizures, thirst, sexual dysfunction, headache, suicidal tendency, organ damage or interactions with anticancer treatments [56, 57]. Many Japanese patients are also hesitant to take psychiatric drugs because of prejudice or stigma [58–60]. It has been reported that Japanese patients with cancer prefer psychotherapy to drug therapy as a treatment for depression, as in previous international studies [12]. The expected advantages of providing BA are as follows: (1) BA focuses on the positive aspects of life’s purpose and value and is accessible and supportive to patients; (2) BA helps patients avoid the side-effects of psychotropic drug therapy; (3) participants will be able to attend BA using techniques that have been scientifically shown to be effective in depression at no cost, with their psychiatric symptoms more fully assessed using a variety of evaluation scales; and (4) participants will be provided with materials on the BA program. Those who score ≤ 23 on the MMSE-J and are found to have cognitive dysfunction will be referred to the psychiatry department at each research facility for appropriate medical treatment.

#### Patient disadvantages and how to minimize them

Since this is a study examining the effects of BA, a behavior-focused psychotherapy, on depression in patients with advanced cancer, we believe that the physical and psychological disadvantages to the study participants will be minimal. However, we will give due consideration to their physical and psychological condition, bearing in mind that they might experience stress due to the content of the intervention program and questionnaires. If exclusion criteria are met during the waiting period, such as worsening of psychiatric symptoms, the patient’s participation will be stopped immediately, and appropriate medical treatment, such as a visit to the psychiatry department of each research facility, will be provided. In addition, patients with a BDI- II score of 21 (moderate) or higher will be enrolled if they wish to participate in the study after being fully informed that they might benefit from pharmacotherapy and the study will be terminated immediately and they will be switched to regular psychiatric care if they experience worsening of psychiatric symptoms based on the discontinuation criteria.

## Duscussion

An important challenge in clinical oncology is determining which interventions are most acceptable and feasible to treat depression in patients with advanced cancer [11]. Though many studies have demonstrated the effectiveness of BA in depression with a high level of evidence [23–25], no studies have explored the effectiveness of BA in patients with advanced cancer.

This multicenter randomized controlled trial is the first study to assess the effectiveness of BA on depression in patients with advanced cancer. If this study can clarify the effectiveness of BA for patients with advanced cancer, our findings will provide evidence about the effectiveness of BA on depression and provide an intervention option that is acceptable and feasible for the treatment of depression in this population. The results of this study will lead to improved mood and rebuilding to regain life purpose and value in this vulnerable population. However, if the effectiveness of BA is not verified in this study, we will need to consider other approaches acceptable and feasible to treat depression in patients with advanced cancer.

### Trial status

The protocol version number is Ver 1.4, and the date is January 7, 2022. The trial was initiated on January 17, 2022, with 38 subjects randomized by March 31, 2024.

We plan to complete recruitment on March 31, 2025.

## Electronic supplementary material

Below is the link to the electronic supplementary material.

Supplementary Material 1

## Data Availability

The datasets used and/or analyzed during the current study are available from the corresponding author on reasonable request.

## Abbreviations

ANOVA: Analysis of variance
BA: Behavioral activation
BADS-SF: Behavioral Activation for Depression Scale-Short Form
BDI-II: Beck Depression Inventory-II
CBT: Cognitive behavioral therapy
ECOG: Eastern Cooperative Oncology Group
FACT-G: Functional Assessment of Cancer Therapy-General
FACIT-Sp-12: Functional Assessment of Chronic Illness Therapy - Spiritual Well-Being 12 Item Scale
GAD-7: Generalized Anxiety Disorder-7
ID: Identification number
IPOS: Integrated Palliative care Outcome Scale
NRS: Numerical rating scale
POS: Palliative Care Outcome Scale
PS: Performance status
QOL: Quality of life
UMIN INDICE: University Hospital Medical Information Internet Data and Information System for Clinical and Epidemiological Research
VQ: Valuing Questionnaire
VQ-O: VQ Obstruction
VQ-P: VQ Progress
