# Supplementary material for: Behavioral activation for depression in patients with advanced cancer: study protocol for a multicenter randomized controlled trial
Source: BMC Cancer. 2023 May 11;23:427. doi: 10.1186/s12885-023-10926-y (PMC10173594; doi:10.1186/s12885-023-10926-y)
Supplement: Supplementary file 1 — Supplementary Material 1 [file 12885_2023_10926_MOESM1_ESM.docx]

**(Original documents in Japanese)**

**行動活性化療法**

**～日々の充実感やよろこびを取り戻すプログラム～**

**多施設共同無作為化比較試験　参加のお願い**

**はじめに**

　この説明文書は、「進行がん患者の抑うつに対する行動活性化療法プログラムの効果検証に関する研究：パイロット・多施設共同無作為化比較試験」について説明したものです。現在抑うつ症状（気持ちの落ち込みや意欲の低下など）がある進行がん患者さんを対象としています。担当医師、および調査者による説明を補い、あなたが研究の内容をより深く理解していただくために用意しました。お読みになって不明な点、疑問がありましたら、担当医師、調査者、または研究事務局まで遠慮なくお尋ねください。

**1．臨床研究について**

　私たちは患者さんに対し、最良のケア・治療を提供すると共に、さらに効果の優れたケア・治療法の開発を試みています。新しく開発された方法が患者さんに対して有用かどうかを実際の患者さんに参加していただいて検討することを「臨床研究」といいます。今後のケア・治療方法の進歩において臨床研究は不可欠なものです。

今回、あなたに紹介させていただく研究は、行動活性化療法の有用性を検討するため、私たちの研究班（研究代表者：国立がん研究センター中央病院精神腫瘍科　平山貴敏）が多施設共同研究として行っているものです。

**2．本研究の目的について**

　行動活性化療法は、これまで抑うつ症状に対して有効であることが示されています。また、行動活性化療法は、多くの患者さんにとって取り組みやすい心理療法です。海外では、がん患者さんの抑うつ症状に対しても有効であることが報告されています。しかし、日本国内においてはがん患者さんに対する研究は十分には行われておらず、日本でも同じ方法で行動活性化療法が役に立つかを検討することが必要とされます。そのため、本研究では、各共同研究実施機関を受診されている患者さんにご協力をいただいて、日本における行動活性化療法の有用性を多施設で調査することを目的としています。

**3．行動活性化療法について**

行動活性化療法は、患者さんが病気の不安や心配にとらわれ、病気になる前よりも閉じこもりがちな生活になっていることに着目し、患者さんがどのような生活を送っていきたいかを大切にしながら過ごすことでその悪循環を断ち切り、日々の充実感やよろこびをもう一度取り戻すという心理療法のプログラムです。その時の気分や体調で過ごし方を決めるのではなく、本当はどのような生活を送っていきたいかを一緒に話し合っていき、具体的にできそうなことから行動に移してもらいます。この一連のプログラムは、行動面から日々の生活を工夫することで患者さんの気持ちのつらさを和らげる手助けとなり、生活の質が向上することが期待されます(図-1を参照)。


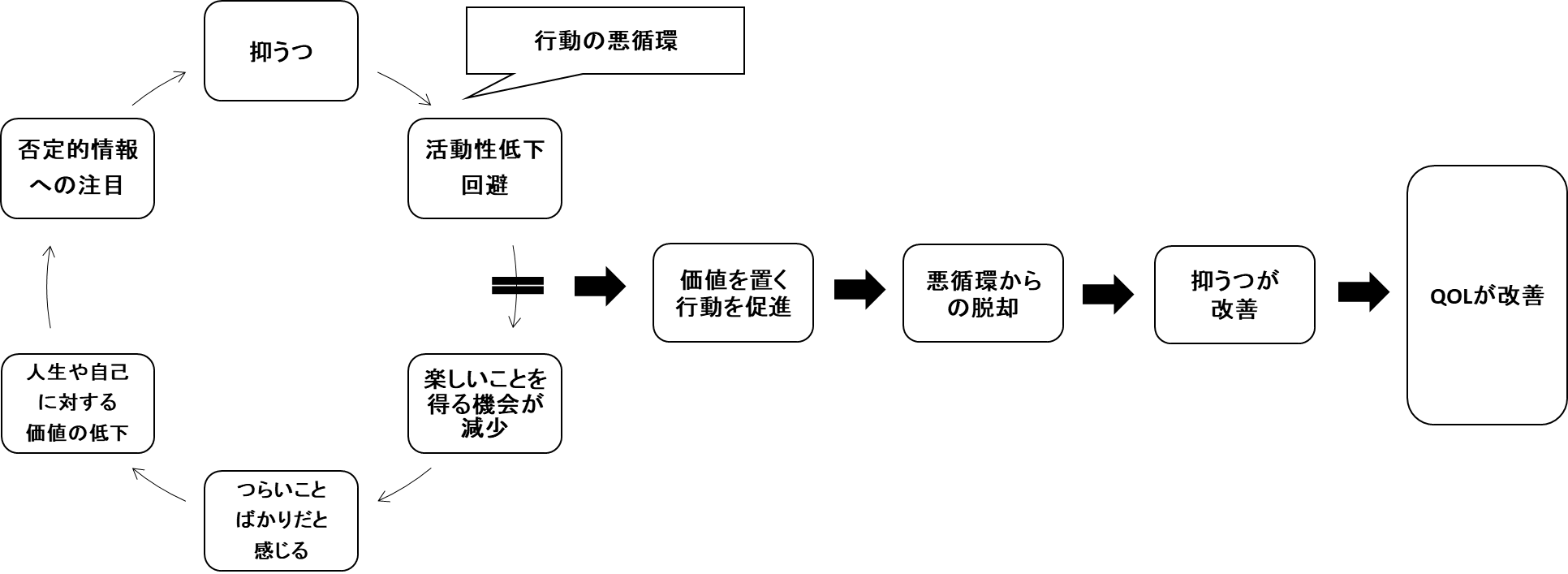


**【図-1 行動活性化療法で抑うつ症状が改善する仕組み】**

**【調査方法】**

あなたがこの研究することに同意いただけましたら、行動活性化療法プログラムを行う介入群あるいは待機群（おおよそ4か月程度待機した後にプログラムを実施）のどちらかになります。どちらになるかは、あなた自身の希望や担当医の判断ではなく「ランダム化」という方法で、コンピューターを使って五分五分の確率でどちらかのグループに決まります。この方法は調べたい治療法以外の条件(性別、年齢、身体や病気の状態など)をほぼ同じにしたグループに分けて比べることで調べたい治療法が本当によいかどうかを比べることができるため、もっとも科学的で良い方法とされています。

プログラムを行う場合、【図-2 調査基本スケジュール】のように初めにアンケートを行います。その後、患者さんの都合の良い日程で１回目のプログラムを行い、その後１～２週に１回の頻度で計7回行います。診療日に合わせることも、別の日に行うことも可能ですので担当者にご相談ください。各セッション（面接）の間隔が1カ月以上空いてしまうと、研究から外れてしまいますのでご理解・ご協力の程よろしくお願いします。

プログラムは、行動活性化療法について訓練を受けた各共同研究実施機関の精神科医、心理療法士が担当者となり行います。全てのプログラムが終了しましたら、終了時、４カ月後にアンケートを行い調査が終了します。

本研究終了時に、精神科医あるいは心理師が診察を行い、抑うつ（気持ちの落ち込み）の治療を継続する必要があるかどうかの判断を行います。治療が必要な場合は、ご相談の上、各研究実施機関の精神科で治療を継続いたします。

尚、研究期間は研究許可日から2025年3月31日までとします。

**【図-２　調査基本スケジュール】**

**4．本研究に参加された患者さんの予測される利益、不利益**

**利益：**海外で科学的に効果の示されているものと同じ方法で、行動活性化療法を費用負担なしで受けることができ、行動活性化療法に関する資料が提供されます。また、様々なアンケートを用いて精神症状を詳しく把握できることで、治療に役立てることができます。日本人のがん患者さんを対象として行動活性化療法を行った場合のデータはまだ十分ではありませんが、海外と同様の効果が期待されています。

**不利益：** 本研究では、患者さん自身へのアンケートをお願いしています。また、行動活性化療法では1～2週に1回50分程度の会話と、毎日5-10分程度のホームワークを行います。そのため、アンケートの回答や会話、ホームワークが身体的な負担になる可能性が考えられます。これらへの対応として、身体的負担に関しては患者さんの様子をみながら慎重に進め、研究参加が負担にならないように十分な配慮を行います。プライバシーの保護に関しては、行動活性化療法で得られた内容は、本研究の目的外で使用されることなく、患者さんが特定されることができないように個人情報部分を改編して厳重に保管されます。

　待機群の場合、おおよそ4か月程度お待ちいただいた後に行動活性化療法を受けていただきます。待機中に体調が変わり薬を使った治療の必要性が生じた場合は、ご希望があれば本研究ではなく、別途行動活性化療法プログラムと同等のプログラムを提供いたします。待機中に不安なことがございましたら遠慮なくご相談ください。

　介入群の場合も、緊急的な使用としての一時的な頓用使用ではない薬を使った治療の必要性が生じた場合は、速やかに適切な治療につなげます。

**5．本研究にかかる費用について**

　本研究費に関して、交通費含め協力費等の支払いや補償はありませんが、研究に参加することにより診療費用が高くなったり安くなったりすることもありません。尚、本研究中に有害事象が発生した場合に治療費用については、健康保険の範囲で自己負担となることをご了承ください。

**6．倫理委員会および各機関の長の承認を受けていること**

　本研究は、国立がん研究センター研究倫理審査委員会で審査され、承認を受け、各機関の長の許可を受けています。

**7．研究参加により予想される利益と不利益**

本研究へ参加することにより、治療上の利益を得る可能性があります。また、新しい治療法の確立に貢献することができます。

ただし、研究で行う治療法は安全性や有効性の評価が定まっていません。そのため、予想より効き目が高くない可能性や、副作用が強く出る可能性、この文書に記載のない副作用が出る可能性もあります。

副作用には血液検査や診察でわかるものと、自覚症状として表れるものがあります。自覚症状はあなたご自身にしかわかりません。我慢せずに体調の変化は、小さなことでも担当医にご相談ください。担当医が治療等適切な処置をいたします。

**8．本研究に参加された場合でも、随時これを撤回できること**

　本研究は、参加される患者さんの自由意思を尊重して行われますので、どのような状況にあっても、参加の同意を撤回することができます。お申し出は、口頭でプログラム担当者、または評価担当者までお願いいたします。

**9．プライバシーの保護について**

　この臨床研究に参加されますと、個人情報と診療情報に関する記録の一部は、共同研究実施機関である国立がん研究センター中央病院、国立がん研究センター東病院、東北大学病院、早稲田大学に提供され保管されます。情報を提供する各機関とのやり取りの際には容易に個人を特定できないように、あなたのお名前ではなく研究登録番号を使用します。研究登録番号はその後に行われる調査の際、担当医が転勤した場合でも、臨床研究に参加していただいているあなたの情報を適切に管理するため、大変重要な情報となります。当院と情報を提供する機関では、これらの情報が外部にもれたり、臨床研究の目的以外に使われないよう最大の努力をしています。この臨床研究にご参加いただける場合は、これらの個人情報の使用につきましてご了承くださいますよう、お願い申し上げます。

**10．外部への情報の提供と将来的な別の研究への利用**

データの取りまとめ機関であるが早稲田大学人間科学学術院内のデータセンターへのデータの提供は、特定の関係者以外がアクセスできない状態で行います。対応表は、各施設の研究責任者が保管・管理します。

本研究で収集した情報（「10. プライバシーの保護について」に記載した、個人が特定できない状態に処理したID化されたデータのみ）は、本研究の目的と関連が深い別の研究に将来的に利用することもあります。具体的には、本研究と同様に抑うつ症状（気持ちの落ち込みや意欲の低下など）がある進行がん患者さんを対象としてより多くの参加者を対象として、行動活性化療法プログラムを行う介入群あるいは待機群を比較する研究を検討しています。

本研究で得られた情報を将来の研究のために使うことに同意いただけた方は、研究終了後も当院で大切に保管させていただきます。また、同意いただけた方でも将来的に別の研究への利用を拒否したい場合は、いつでも申し出ることで同意を撤回することができます。新たな研究に用いる際には、あらためて研究計画書を作成し、研究倫理審査委員会の承認を受けた上で行います。

責任を有する者の氏名及び所属研究機関

研究責任者：平山 貴敏

連絡先 ：国立研究開発法人国立がん研究センター中央病院 　精神腫瘍科

〒104-0045 東京都中央区築地 5-1-1

Tel: 03-3542-2511（代）

**11．****研究に関する資料等の利用と保存**

本研究に関する原資料は研究終了後から5年間、または最終公表についての報告日から3年経過した日のいずれか遅い日まで保管します。

**12．研究組織・連絡先**

この研究について不明な点や心配に思うことがあれば、いつでも遠慮なく担当医師にお尋ねください。担当医師に聞きづらいことや、この研究の責任者に直接お尋ねになりたいことがある場合は、下記の「研究事務局」または、各施設の問い合わせ窓口までお問い合わせください。

担当医/研究担当者：平山 貴敏

連絡先 ：国立研究開発法人国立がん研究センター中央病院 　精神腫瘍科

〒104-0045 東京都中央区築地 5-1-1

Tel: 03-3542-2511（代）

研究代表者/研究事務局（連絡窓口）

国立がん研究センター中央病院　精神腫瘍科

　　住所　〒104-0042　東京都中央区築地5-1-1

　　電話番号　03-3542-2511　内線7111

　　担当　平山　貴敏

各施設の問い合わせ窓口

（共同研究機関名・各機関の研究責任者名及び役割、連絡先の追記をお願いします）

① 国立がん研究センター東病院　精神腫瘍科　研究責任者　氏名 小川　朝生

住所　〒277-8577　千葉県柏市柏の葉6-5-1

電話番号　04-7133-1111

② 東北大学病院　精神科　研究責任者　氏名　五十嵐　江美

住所　〒980-8574　仙台市青葉区星陵町1-1

電話番号　022-717-7262

③ 早稲田大学人間科学学術院　研究責任者（データ管理者）氏名　鈴木　伸一

住所　〒359-1192　所沢市三ケ島2-579-15

電話番号　04-2947-6955

**13．本研究の資金源**

　科学研究費助成事業（科学研究費補助金）基盤研究(B)（一般）（研究課題名：進行がん患者への新たな心理的支援法開発に向けた行動活性化療法のランダム化比較試験、研究代表者：早稲田大学人間科学学術院教授・国立がん研究センターがん対策研究所 客員研究員　鈴木伸一）を資金源とします。

**14．利益相反（COI）の管理について**

　本研究に関して開示する利益相反はありません。本研究における利益相反の管理は、各研究実施機関が、当該施設所属の研究者に関して行っています。

**15．文書による同意**

　本研究では、患者さんの同意を文書で得ることが規定されています。研究の内容を十分に理解し納得された上で、研究への参加に同意していただけるのであれば、同意書にご署名および日付の記入をお願いいたします。

**(Translation into English)**

Behavioral Activation:

A Program to Restore a Daily Sense of Fulfillment and Joy

Request for Participation in a Multicenter Randomized Controlled Trial

**INTRODUCTION**

This informational document describes the "Study on the effectiveness of a behavioral activation program for depression in patients with advanced cancer: a multicenter randomized controlled trial." Patients with advanced cancer who currently have depressive symptoms (e.g., depressed mood or low motivation) are eligible to participate this study. To help you better understand this study, we have prepared this information to supplement the explanations provided by your physician and the program practitioner. If you have any questions or concerns after reading this document, please do not hesitate to ask your physician, the program practitioner, or the research secretariat.

**1. CLINICAL STUDY**

We attempt to provide the best possible treatment and care to our patients while at the same time developing more effective methods of treatment and care. A clinical study examines whether a newly developed method is useful for patients or not by having actual patients participate in the study. Clinical studies are essential to advancement of medicine and treatment methods.

This study is being conducted by our study group (Research representative: Takatoshi Hirayama, Department of Psycho-Oncology, National Cancer Center Hospital) as a collaborative multicenter study to investigate the efficacy of behavioral activation.

**2. PURPOSE OF THIS STUDY**

Behavioral activation has been shown to be effective for depressive symptoms. Behavioral activation is a type of psychotherapy that is easy for many patients to undergo. It has been reported to be effective for depressive symptoms in patients with cancer overseas. However, there are not enough studies of patients with cancer in Japan. Thus, it is necessary to investigate whether behavioral activation is useful in the same way in Japan. The purpose of this study is to investigate the efficacy of behavioral activation in Japan at multiple institutions with the cooperation of patients who visit each collaborating research institution.

**3. BEHAVIORAL ACTIVATION**

Behavioral activation focuses on the fact that patients tend to be anxious and worried about their illness and tend to live more confined lives than before they became ill. This psychotherapy program aims to break this vicious cycle and restore a sense of daily fulfillment and joy by focusing on the kind of life the patient wants to lead. Instead of deciding how to spend your time based on your current mood or physical condition, we will discuss with you what kind of life you really want to lead. We will ask you to start with specific things you can do and then move on to behavior. This program is expected to alleviate mental distress and improve quality of life by modifying daily life via a behavioral aspect (Please refer to Figure 1).

Vicious cycle of behavior

Figure 1. How behavioral activation improves depressive symptoms.

QOL, quality of life.

**【STUDY METHODS】**

If you agree to enroll in this study, you will be either in the intervention group, which will include the behavioral activation program, or in the waiting group, which will wait approximately 4 months before receiving behavioral activation. Which group you will be in is not determined by your own wishes or by your physician. It will be determined by a method called randomization. A computer is used to determine which group you will be in with a 50-50 chance of being in either group. This method is considered the best and most scientific method because it allows us to compare whether the treatment we want to examine is really good or not by dividing the patients into groups with almost the same characteristics (gender, age, physical and disease status, etc.) other than the treatment we want to examine.

A questionnaire will be given at the beginning of the program, as shown in Figure 2 (Basic Survey Schedule). Next, the first program session will be conducted on a schedule that is convenient for you, followed by a total of seven sessions, once every 1 to 2 weeks. It can be conducted in conjunction with a clinic day or on a different day; consult with the person in charge. If there is an interval of more than 1 month between sessions (interviews), you will be excluded from this study. We appreciate your understanding and cooperation.

The program sessions are conducted by psychiatrists and psychotherapists at each collaborating institution who are trained in behavioral activation. You will receive a questionnaire after completion of all program sessions and 4 months later.

At the end of this study, a psychiatrist or psychologist will examine you to determine if treatment for depression (feeling down) needs to be continued. If treatment is necessary, we will consult with you and continue treatment at the psychiatry department of each collaborating research institution.

This study period starts on the date of approval (January 17, 2022) and ends on March 31, 2025.

Intervention group

Pre-interview

Questionnaire

Questionnaire after the program ends

Questionnaire 4 months later

**Behavioral activation**

**program**

Control group

Pre-interview

Questionnaire

Questionnaire 4 months later

Questionnaire after the program ends

Before beginning

Questionnaire

Wait

(Approximately 4 months)

**Behavioral activation**

**program**

Figure 2. Basic survey schedule

**4. PROJECTED BENEFITS AND DISADVANTAGES FOR PATIENTS WHO PARTICIPATE IN THIS STUDY**

**BENEFITS:** The program provides access to behavioral activation at no cost, using the same methods that have been scientifically shown to be effective abroad. It also provides materials on behavioral activation. Although there is currently insufficient data on behavioral activation in Japanese patients with cancer, it is expected to be as effective as in other countries.

**DISADVANTAGES:** In this study, patients will be asked to complete a questionnaire about themselves. In addition, behavioral activation involves 50 minutes of conversation every 1–2 weeks and 5–10 minutes of homework daily. Therefore, it is possible that the questionnaire responses, conversations, and homework might become a physical burden. To cope with these issues, we will proceed with caution while monitoring the patient's physical condition. We will give sufficient consideration so that participation in this study does not become a burden. With regard to the protection of privacy, the information obtained for the behavioral activation will not be used for any purpose other than this study. Information will be kept in strict confidence, with the personal information portion edited so that patients cannot be identified.

If you are assigned to the waiting group, you will receive the behavioral activation after a waiting period of approximately 4 months. If your condition changes during the waiting period and a need for treatment with medication arises, we will provide you with a separate program equivalent to the behavioral activation program instead of study participation if you wish. Please do not hesitate to contact us if you have any concerns during the waiting period.

If you are assigned to the intervention group, and the need arises for treatment with a drug that is not for temporary, emergency use, you will be promptly referred for appropriate treatment.

**5. COST OF THIS STUDY**

There are no payments or compensation for participation, including transportation expenses, in relation to this study. Participating in this study will not change medical expenses. We would like to ask for your understanding that you will be responsible for the cost of treatment for any adverse events that occur during this study within the scope of your health insurance.

**6. APPROVED BY THE ETHICS COMMITTEE AND THE HEADS OF EACH INSTITUTION**

This study was reviewed and approved by the National Cancer Center Institutional Review Board, Japan. It was approved by the head of each collaborating research institution.

**7. ANTICIPATED BENEFITS AND DISADVANTAGES OF PARTICIPATION IN THIS STUDY**

Participation in this study might provide therapeutic benefits. It can also contribute to the establishment of new treatment methods. However, the safety and efficacy of the treatments in this study have not been established. Therefore, there is a possibility that they might not be as effective as expected. There might be stronger side effects or side effects not described in this document. Some side effects can be detected with blood tests or medical examinations, while others are subjective symptoms. Only you can be aware of subjective symptoms. Please do not hesitate in consulting your physician about any changes in your physical condition, even if the changes are small. Your physician will take appropriate measures, which might include treatment.

**8. VOLUNTARINESS OF PARTICIPATION IN THIS STUDY AND YOUR FREEDOM TO WITHDRAW**

Since this study is conducted with respect of the free will of the participating patients, patients can withdraw their consent to participate at any time. If you wish to withdraw your consent, please make your request verbally to the program practitioner or outcome assessor.

**9. PROTECTION OF PRIVACY**

When you participate in this clinical study, some of your personal information and medical records will be provided to and stored by the National Cancer Center Hospital, National Cancer Center Hospital East, Tohoku University Hospital, and Waseda University, which are the collaborating research institutions. We will use your study registration number instead of your name so that we cannot easily identify you when we communicate with the institutions providing the information. The study registration number is very important for the proper management of information in subsequent investigations, even if your physician has been transferred. We and the collaborating research institutions to which we provide information will make every effort to ensure that this information is not disclosed to outside parties or used for purposes other than this clinical study. If you participate in the clinical study, please understand the use of this personal information.

**10. PROVISION OF INFORMATION TO EXTERNAL PARTIES AND USE FOR FUTURE STUDIES**

Data will be provided to the data center of the Faculty of Human Sciences, Waseda University, which is responsible for data collection. It will be provided in a format where only certain parties can access the data. The correspondence table is stored and managed by the research director of each institution.

Information collected in this study (only ID data processed in a non-personally identifiable manner as described in Section 9 (Protection of Privacy)) might be used for other studies in the future that are closely related to the objectives of this study. Specifically, we are considering a study comparing an intervention group to a wait-list control group in which a behavioral activation program is conducted with a large number of participants with advanced cancer who have depressive symptoms (e.g., depressed mood or low motivation) similar to those in this study.

If you agree to allow us to use the information obtained in this study for future studies, the information will be kept in our clinic after this study has been completed. Even if you have given your consent, you may withdraw your consent at any time if you wish to refuse the use of your data for other study in the future. When using the data for a new study, we will prepare a new study plan and obtain the approval of the Study Ethics Review Committee.

Name of the responsible person and the affiliated research institution

Research representative: Takatoshi Hirayama

Contact information: Department of Psycho-Oncology, National Cancer Center Hospital

5-1-1, Tsukiji, Chuo-ku, Tokyo, 104-0045, Japan

Tel: 03-3542-2511

**11. USE AND PRESERVATION OF MATERIALS RELATED TO THE STUDY**

Original materials related to this study will be retained for 5 years after the completion of this study or until 3 years have elapsed from the date of the final publication, whichever is later.

**12. RESEARCH INSTITUTE AND CONTACTS**

If you have any questions or concerns about this study, please do not hesitate to ask your physician at any time. If you have any questions that you do not feel comfortable asking your physician or would like to ask the person responsible for this study directly, please contact the research secretariat listed below or the contact person at each institution.

MD / Researcher: Takatoshi Hirayama

Department of Psycho-Oncology, National Cancer Center Hospital

Address: 5-1-1, Tsukiji, Chuo-ku, Tokyo, 104-0045, Japan

Tel: 03-3542-2511

Research representative / Research secretariat (Contact): Takatoshi Hirayama

Department of Psycho-Oncology, National Cancer Center Hospital

Address: 5-1-1, Tsukiji, Chuo-ku, Tokyo, 104-0045, Japan

Tel: 03-3542-2511 Extension number: 7111

Contact for inquiries at each institution:

1. Research representative: Asao Ogawa

Department of Psycho-Oncology, National Cancer Center Hospital East

Address: 6-5-1, Kashiwanoha, Kashiwa-city, Chiba, 277-8577, Japan

Tel: 04-7133-1111

2. Research representative: Emi Igarashi

Department of Psychiatry, Tohoku University Hospital

Address: 1-1, Seiryo-machi, Aoba-ku, Sendai, Miyagi, 980-8574, Japan

Tel: 022-717-7262

3. Research representative (Data manager): Shin-ichi Suzuki

Faculty of Human Sciences, Waseda University

Address: 2-579-15 Mikajima, Tokorozawa-city, Saitama, 359-1192, Japan

Tel: 04-2947-6955

**13. SOURCES OF FUNDING FOR THIS STUDY**

This study is funded by a Grant-in-Aid for Scientific Research and a Grant-in-Aid for Scientific Research, Basic Research (B) (General) titled “Randomized controlled trial of behavioral activation for the development of a new psychological support method for advanced patients with cancer.” The research representative is Shin-ichi Suzuki, Professor, Faculty of Human Sciences, Waseda University and Visiting Researcher, National Cancer Center Research Institute.

**14. CONFLICT OF INTEREST MANAGEMENT**

There are no conflicts of interest to disclose for this study. Conflicts of interest in this study are managed by each research institution with respect to the researchers affiliated with that institution.

**15. DOCUMENTED CONSENT**

Witten consent for participation in this study will be obtained from patients. If you fully understand and agree to participate in this study, please sign and date the consent form.
